# Roles of Phosphate Recognition in Inositol 1,3,4,5,6-Pentakisphosphate 2-Kinase (IPK1) Substrate Binding and Activation*

**DOI:** 10.1074/jbc.M113.487777

**Published:** 2013-07-24

**Authors:** Varin Gosein, Gregory J. Miller

**Affiliations:** From the ‡ Department of Pharmacology and Therapeutics, McGill University, Montréal, Québec H3G 1Y6, Canada,; the § Groupe de Recherche Axé sur la Structure des Protéines, McGill University, Montréal, Québec H3G 0B1, Canada, and; the ¶ Department of Chemistry, The Catholic University of America, Washington, D.C. 20064

**Keywords:** Enzyme Mechanisms, Inositol Phosphates, Isothermal Titration Calorimetry, Ligand-binding Protein, Phosphorylation, IP_6_, IPK1, Kinase Activation, Substrate Recognition

## Abstract

Inositol phosphate kinases (IPKs) sequentially phosphorylate inositol phosphates (IPs) to yield a group of small signaling molecules involved in diverse cellular processes. IPK1 (inositol 1,3,4,5,6-pentakisphosphate 2-kinase) phosphorylates inositol 1,3,4,5,6-pentakisphosphate to inositol 1,2,3,4,5,6-hexakisphosphate; however, the mechanism of IP recognition employed by IPK1 is currently unresolved. We demonstrated previously that IPK1 possesses an unstable N-terminal lobe in the absence of IP, which led us to propose that the phosphate profile of the IP was linked to stabilization of IPK1. Here, we describe a systematic study to determine the roles of the 1-, 3-, 5-, and 6-phosphate groups of inositol 1,3,4,5,6-pentakisphosphate in IP binding and IPK1 activation. The 5- and 6-phosphate groups were the most important for IP binding to IPK1, and the 1- and 3-phosphate groups were more important for IPK1 activation than the others. Moreover, we demonstrate that there are three critical residues (Arg-130, Lys-170, and Lys-411) necessary for IPK1 activity. Arg-130 is the only substrate-binding N-terminal lobe residue that can render IPK1 inactive; its 1-phosphate is critical for full IPK1 activity and for stabilization of the active conformation of IPK1. Taken together, our results support the model for recognition of the IP substrate by IPK1 in which (i) the 4-, 5-, and 6-phosphates are initially recognized by the C-terminal lobe, and subsequently, (ii) the interaction between the 1-phosphate and Arg-130 stabilizes the N-terminal lobe and activates IPK1. This model of IP recognition, believed to be unique among IPKs, could be exploited for selective inhibition of IPK1 in future studies that investigate the role of higher IPs.

## Teaser

**Background:** The mechanism of substrate recognition for IPK1 (inositol 1,3,4,5,6-pentakisphosphate 2-kinase) is unresolved.

**Results:** Binding and activity data reveal specific roles for each phosphate of IP_5_.

**Conclusion:** The phosphate profile of IP_5_ is mechanistically critical to IPK1 activation.

**Significance:** Identifying determinants of substrate specificity will aid in the design of selective inhibitors for IPK1.

## Introduction

Inositol phosphates (IPs)^2^ are a group of small molecules that play critical roles in cellular signaling (1). IP signaling regulates DNA editing and repair (2), vesicle transport (3), and ion channel regulation (4) and has been implicated in diseases such as cancer and diabetes (5). IPs are produced by sequential phosphorylation of inositol 1,4,5-trisphosphate by a family of enzymes known as IP kinases (IPKs) (1). Similarity between IPs, which sometimes differ by only one phosphate group on the inositol ring, demands that IPKs use mechanisms to recognize and phosphorylate specific positions of their IP substrates while excluding highly similar molecules. Crystal structures from each of the IPK subfamilies have revealed that the structural determinants for IP discrimination vary between IPKs. IP3K (Inositol 1,4,5-trisphosphate 3-kinase) employs shape complementarity to recognize precisely positioned phosphate and hydroxyl groups of inositol 1,4,5-trisphosphate (6). In contrast, ITPK1 (inositol 1,3,4-trisphosphate 5/6-kinase/inositol 3,4,5,6-tetrakisphosphate 1-kinase) discriminates among IPs using phosphate affinity and stereochemical features to establish contacts with phosphates that are sufficient for substrate recognition (7). Crystal structures of IPK1 (inositol 1,3,4,5,6-pentakisphosphate 2-kinase) in its IP substrate- and product-bound forms reveal extensive contacts with all phosphate groups of the bound IPs (8). These structures reveal how inositol 1,3,4,5,6-pentakisphosphate (IP_5_) is phosphorylated on its axial 2′-hydroxyl, yielding inositol 1,2,3,4,5,6-hexakisphosphate, but they do not suggest a mechanism through which IPK1 selectively recognizes IP_5_ as its substrate while excluding other highly phosphorylated IPs with free axial 2′-hydroxyl groups. We recently determined the crystal structure of wild-type IPK1 in an IP-free state, which exhibited disorder within its N-terminal lobe (N-lobe) of the kinase, centered at Arg-130 (9). This IP-free structure suggests that binding of IP substrate plays a role in stabilization of the N- and C-lobes of the kinase, which is an important step in the activation of protein kinases (10–12).

Our current objective was to define the contributions of the individual phosphate groups of the IP to binding and to recognition of bound IP as a substrate. The results demonstrate that each phosphate group of the IP plays a different role in binding and activation of IPK1 and that there are three critical contacts formed between IPK1 and the IP that mediate IPK1 activation.

## EXPERIMENTAL PROCEDURES

### 

#### 

##### Generation of Alanine Mutants

Residues that interact with IP_5_, either directly or through solvent molecules, were identified using previous crystal structures (9). Mutation of these residues to alanine was performed by site-directed mutagenesis using the QuikChange method (Stratagene). A pET28a vector containing wild-type *Arabidopsis thaliana* IPK1 and a hexahistidine tag was used as a template (a kind gift from Dr. C. A. Brearley, University of East Anglia). All mutations were verified by DNA sequencing.

##### Protein Expression and Purification

Wild-type IPK1 and alanine mutants were expressed in BL21-AI cells (Invitrogen) that were grown in Terrific Broth to *A*_600_ = 1.5 and induced with 0.5 mm isopropyl β-d-thiogalactopyranoside and 0.1% l-arabinose at 18 °C for 20 h. Cells were lysed in 10 mm Tris-HCl (pH 8.0), 250 mm NaCl, and 50% glycerol using a sonicator. The supernatant was separated from the lysate by centrifugation at 45,000 × *g*. The supernatant was then diluted 5-fold using 20 mm Tris-HCl (pH 8.0) and 500 mm NaCl, and 25 mm imidazole was added. IPK1 was purified using nickel-nitrilotriacetic acid beads (Thermo Scientific) in a gravity column using 4 ml of dry beads/250 ml of culture. The beads were washed with 20 column volumes of 50 mm KPO_4_ (pH 8.0), 800 mm NaCl, 1% Triton X-100, and 1.7 mm β-mercaptoethanol. Protein was eluted using 250 mm imidazole in 20 mm Tris-HCl (pH 8.0) and 300 mm NaCl, and then 2 mm DTT was added to the eluate. The protein concentration was determined by Bradford assay (Thermo Scientific) using BSA as a standard. Protein was stored at 4 °C and used within 72 h.

##### Isothermal Titration Calorimetry (ITC)

Experiments were performed on a MicroCal iTC200 titration calorimeter (GE Healthcare). Wild-type IPK1 was purified and dialyzed into ITC buffer containing 50 mm HEPES (pH 7.5), 6 mm MgCl_2_, 150 mm NaCl, and 1 mm tris(2-carboxyethyl)phosphine (pH 7.0). After protein dialysis was complete, dialysis buffer was used to dissolve the ligands, IP, and AMP-PNP (Jena Bioscience). Titration experiments were performed at 25 °C with 100 μm IPK1 and 1 mm AMP-PNP in the cell and 1–2 mm IP in the syringe to ensure a final IP:IPK1 molar ratio of at least 2:1. Titration experiments were performed at least twice for each IP, and one set was chosen to represent data. Calorimetric data were analyzed using Origin 7.0 (MicroCal). Data were fitted with a one-site model using Equation 1,

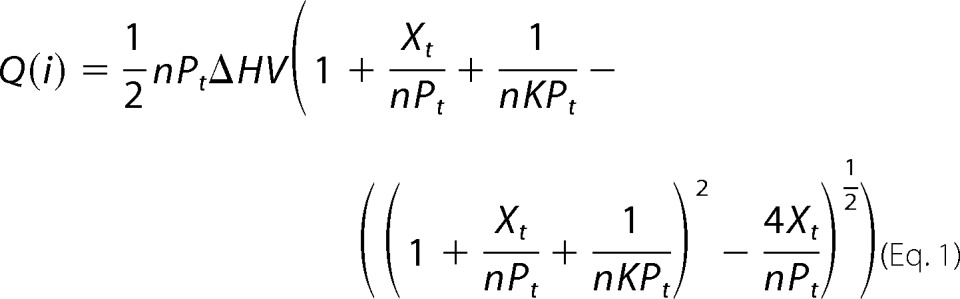
 where *n* is the number of IP-binding sites on IPK1, *P_t_* is the total concentration of IPK1, *X_t_* is the total concentration of IP, *V* is the cell volume, *K* is the binding constant, and Δ*H* corresponds to the enthalpy change due to IP-IPK1 binding. The heat corresponding to the *i*th injection only, Δ*Q*(*i*), is equal to the difference between *Q*(*i*) and *Q*(*i* − *1*) and is given by Equation 2,

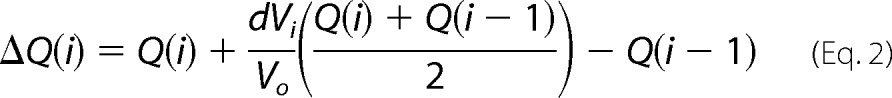
 which is corrected by the injection volume (d*V_i_*) for the displaced volume.

##### IPK1 Activity Assay

IPs (IP_5_, 1,3,4,5-inositol tetrakisphosphate (IP_4_), 1,4,5,6-IP_4_, 1,3,4,6-IP_4_, and 3,4,5,6-IP_4_) were purchased from Cayman Chemical Company. A source for 1,3,5,6-IP_4_ was not located. IPK1 kinase activity was assessed using the Kinase-Glo Max luminescent kinase assay (Promega) following the manufacturer's instructions. Kinase reactions were performed in 25-μl volumes on black 96-well plates at 25 °C. The reaction mixture contained 50 mm HEPES (pH 7.5), 6 mm MgCl_2_, 50 mm NaCl, and 300 μm ATP. 25 μl of Kinase-Glo reagent was added to stop the reaction, and luminescence was measured after 20 min on a Berthold Orion II microplate luminometer. Initially, 80 μm IP was used, and the amount of enzyme was varied to determine conditions in which product formation was linear over 30 min. Subsequently, an array of reactions with varying concentrations of IP (20, 40, 60, 80, 100, 120, and 140 μm) stopped at various time points (2, 5, 10, 20, and 30 min) were performed in triplicate. The rate of product formation *versus* IP concentration was plotted and fitted to the Michaelis-Menten equation using nonlinear regression to determine *K_m_* and *V*_max_ (GraphPad Software). The *k*_cat_ values were calculated using the equation *k*_cat_ = *V*_max_/[*E*], where [*E*] is the micromolar enzyme concentration.

##### IPK1 Alanine Mutant Activity Assay

Initially, 150 ng of each mutant was tested for kinase activity with 80 μm IP_5_ after 30 min. Mutants that exhibited little or no activity were retested using 750 ng of enzyme. Active mutants were further characterized for their kinetic parameters with IP_5_ as a substrate using the abovementioned approach.

## RESULTS

### 

#### 

##### 5- and 6-Phosphates Are Important for Binding

To determine the role of phosphates at each position of the inositol ring, we measured the effect on binding affinity when using IPs lacking a phosphate group at the 1-, 3-, 5-, or 6-position. Using ITC, we obtained *K_D_* values for each IP_4_ and IP_5_ (Table 1). As expected, IP_5_, the native substrate for IPK1, displayed the highest binding affinity, with *K_D_* = 0.60 μm. IP_4_s exhibited a range of binding affinities. The *K_D_* values of 1,4,5,6-IP_4_ and 3,4,5,6-IP_4_ were 13-fold higher than that of IP_5_, whereas the *K_D_* values of 1,3,4,6-IP_4_ and 1,3,4,5-IP_4_ were at least 30-fold higher. These results indicate that different phosphate groups have varying contributions to the binding affinity of IP_5_ for IPK1. Comparison of the IP_4_ *K_D_*:IP_5_ *K_D_* ratios revealed that the 5- and 6-phosphates contributed the most to the binding affinity of the IP, as the absence of either phosphate group dramatically increased the *K_D_* (Table 1).

**TABLE 1:** **Binding data of IPK1 for IP_5_ and IP_4_s**

IP	*N* (sites)	*K*	*K_D_*	Δ*H*	Δ*S*
		*m*^−*1*^	μ*m*	*cal/mol*	*cal/mol/degrees*
IP_5_	0.895	1.68 × 10^6^	0.60	−11,900	−11.4
1,3,4,6-IP_4_	0.895	3.72 × 10^4^	26.88	−8720	−8.34
1,4,5,6-IP_4_	1.14	1.22 × 10^5^	8.20	−7551	−2.05
3,4,5,6-IP_4_	0.656	1.33 × 10^5^	7.52	−18,300	−37.9
1,3,4,5-IP_4_	1.19	5.49 × 10^4^	18.21	−10,140	−12.3

##### 1- and 3-Phosphates Are Important for Substrate Recognition

**FIGURE 1.**
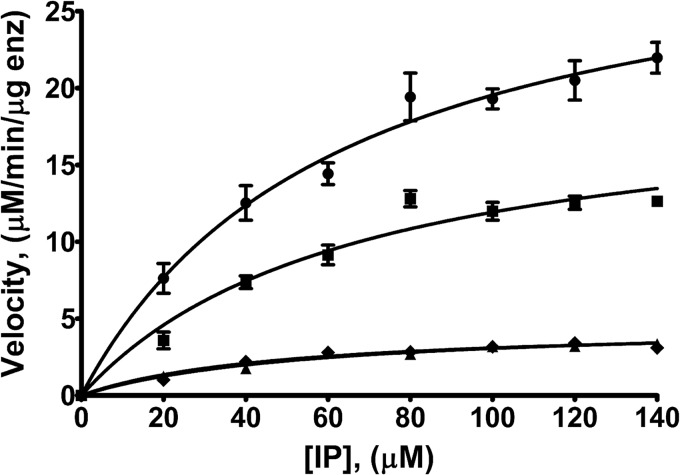
**Kinetic analysis of the kinase activity of IPK1 for IP_5_ and IP_4_s.** IPK1 kinase activity was assessed using a luminescence-based assay. The rate of product formation *versus* IP concentration was plotted and fitted to the Michaelis-Menten equation. Each point represents the mean ± S.D. of triplicate experiments. ●, IP_5_; ■, 1,3,4,6-IP_4_; ♦, 3,4,5,6-IP_4_; ▴, 1,4,5,6-IP_4_. *enz*, enzyme.

We tested the kinase activity of IPK1 for IP_5_ and IP_4_s using a luminescence-based assay to determine which phosphates identify an IP as a substrate for IPK1 (Fig. 1 and Table 2). The kinase activity of IPK1 was maximal in the presence of IP_5_, with *k*_cat_ = 44.02 ± 3.19 nmol/min. With 1,3,4,6-IP_4_, an IP lacking the 5-phosphate, there was a modest decrease in kinase activity, with *k*_cat_ = 27.48 ± 4.00 nmol/min. In contrast, IPs lacking 1- and 3-phosphate groups exhibited a substantial 85% decrease in activity compared with IP_5_. No activity was detected when 1,3,4,5-IP_4_ was used as a substrate, suggesting that the 6-phosphate may also be important for activation. These results indicate that the 1- and 3-phosphates are important for the IP to be recognized as a substrate by IPK1. We also observed that the *K_D_* values for IPs lacking phosphates varied considerably, whereas the *K_m_* for each IP remained nearly constant (Table 2). This disconnect suggests that the kinetic parameters of ligand binding or catalysis change along with the binding affinity, but we cannot define with the current set of assays how they change.

**TABLE 2:** **Kinetic parameters of IPK1 for IP_5_ and IP_4_s** Data represent the mean ± S.D. of triplicate experiments. ND, no activity detected.

IP	*K_m_*	*k*_cat_
	μm	min^−1^
IP_5_	63.05 ± 10.77	44.02 ± 3.19
1,3,4,6-IP_4_	66.81 ± 22.37	27.48 ± 4.00
1,4,5,6-IP_4_	55.99 ± 13.95	6.60 ± 0.66
3,4,5,6-IP_4_	47.54 ± 13.94	6.30 ± 0.68
1,3,4,5-IP_4_	ND	ND

##### Alanine Mutants Identify Active Site Residues Critical for IPK1 Activity

**FIGURE 2.**
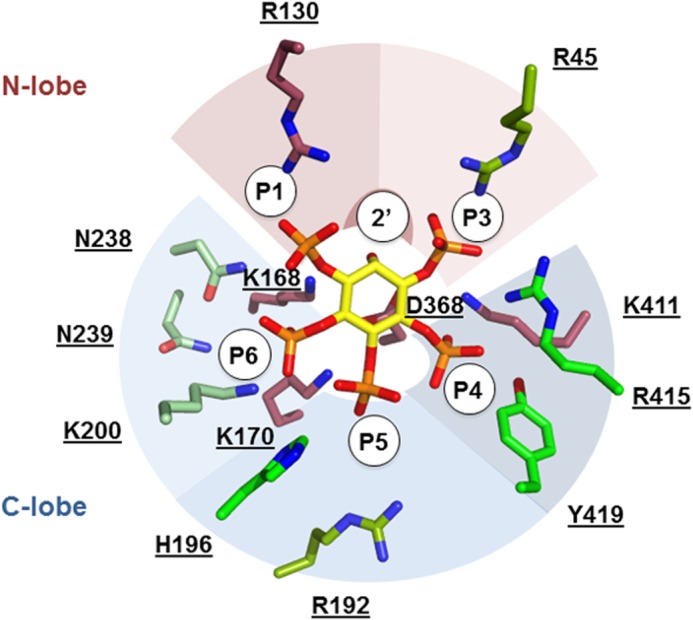
**Structural representation of kinetic parameters of alanine mutants.** IP_5_ is shown in *yellow* stick form. The side chains of IP-binding residues are shown in stick form and colored according to alanine mutant *k*_cat_. *Maroon* indicates no activity. *Darker green shades* indicate reduced activity compared with wild-type IPK1. *Green* indicates equivalent activity to wild-type IPK1. Side chains are grouped according to bound phosphate, overlaid with colored arcs (*red*, N-lobe; *blue*, C-lobe).

To identify the contacts between IPK1 and IP that are essential for activity, we mutated to alanine those IPK1 residues that interact with the IP and tested these mutants for activity for IP_5_ (Table 3). To compare the kinase activity between mutants, we determined *k*_cat_ values for each mutant. For wild-type IPK1, *k*_cat_ = 44.02 ± 3.19 nmol/min. Mutation of Arg-130, the only residue that interacts with the 1-phosphate of the IP, resulted in no detectable activity. Mutations of Lys-168 and Asp-368, which mediate phosphoryl transfer from ATP to the 2′-hydroxyl, also exhibited no activity, as expected. The R45A mutant, which abolished the contact with the 3-phosphate, displayed a 40% decrease in kinase activity (*k*_cat_ = 27.16 ± 1.79 nmol/min), whereas the R415A and Y419A mutants, which abolished contacts with the 4-phosphate, both displayed activity equivalent to wild-type IPK1. In contrast, the K411A mutant, which abolished contacts with both the 3- and 4-phosphates, showed no activity. The R192A mutant displayed a modest decrease in kinase activity (*k*_cat_ = 22.01 ± 2.05 nmol/min), and the H196A mutant had no effect on kinase activity (*k*_cat_ = 34.12 ± 4.33 nmol/min), demonstrating that interactions with the 5-position have modest impact on catalytic activity. The K170A mutant, which abolished interactions with both the 5- and 6-phosphates, showed no activity. Finally, the K200A, N238A, and N239A mutants, which eliminated contacts with the 6-phosphate, displayed reduced activity compared with wild-type IPK1. Fig. 2 summarizes the effect of alanine mutations in the inositide-binding site on IPK1 activity. Here, we observed that the residues that interact with more than one phosphate play important roles in substrate recognition, whereas most residues that interact with a single phosphate play lesser roles in this process.

**TABLE 3:** **Kinetic parameters of IPK1 alanine mutants for IP_5_** Data represent the mean ± S.D. of triplicate experiments. The last column indicates the IP phosphate that interacts with the mutant side chain, either directly or indirectly, through ordered water molecules. ND, no activity detected.

Mutant	*K_m_*	*k*_cat_	PO_4_ interaction
	μ*m*	*min*^−*1*^	
R45A	54.28 ± 9.07	27.16 ± 1.79	3
R130A	ND	ND	1
K168A	ND	ND	2
K170A	ND	ND	5, 6
R192A	59.35 ± 13.41	22.01 ± 2.05	5
H196A	43.48 ± 15.62	34.12 ± 4.33	5
K200A	39.79 ± 19.52	16.74 ± 2.77	6
N238A	33.72 ± 7.918	14.74 ± 1.07	6
N239A	83.30 ± 9.53	15.89 ± 0.87	6
D368A	ND	ND	2
K411A	ND	ND	3, 4
R415A	62.27 ± 21.89	38.71 ± 5.73	3, 4
Y419A	76.86 ± 16.94	41.62 ± 4.25	4

## DISCUSSION

### 

#### 

##### Roles of Different Phosphate Groups: Binding versus Activation

Here, we performed a systematic study to identify the relative contributions of the 1-, 3-, 5-, and 6-phosphates to IP_5_ binding affinity and recognition as an IPK1 substrate. Initially, we used ITC to determine the binding affinity of IPK1 for IP_5_ and for a set of IP_4_s, each lacking a single phosphate group. We observed a spectrum of affinities for these differently phosphorylated IP_4_ molecules, indicating that phosphates contribute differently to binding. IPs lacking the 5- or 6-phosphate displayed the lowest binding affinity for IPK1, whereas IPs lacking the 1- or 3-phosphate displayed only moderately decreased binding affinity (Table 1). The structure of nucleotide-bound IPK1 (Protein Data Bank code 3UDS) revealed the N-lobe to be unstable compared with the IP-bound state, and in a recent structure of IPK1 engineered to crystallize in the absence of IP (Protein Data Bank code 4AXC), the N-lobe was too far away from the C-lobe to form a complete inositide-binding pocket (9, 13). In both wild-type and mutant structures, the N-lobe fails to assemble into the active conformation. Our ITC binding data, which indicate that the C-lobe-binding 5- and 6-phosphates contribute substantially more to binding, are consistent with the C-lobe playing a dominant role in substrate recruitment. This collection of structures and the binding data support the model that substrate recruitment likely occurs though the stable C-lobe, which comprises half of the IP-binding site. To complete assembly of the IP-binding site, IP must bind to both the N- and C-lobes, thereby coupling binding of the substrate to stabilization of the kinase.

We further investigated the contribution of each phosphate group of the IP to its recognition as a substrate and to activation of IPK1. We determined the *K_m_* and *k*_cat_ of IPK1 in the presence of IP_5_ and our series of IP_4_s. These data indicate that the *k*_cat_ for IPK1 is substantially decreased for IPs lacking the 1- or 3-phosphate compared with IP_5_ and 1,3,4,6-IP_4_ (Table 2). Preliminary studies of IPK1 substrate specificity also revealed the use of 1,3,4,6-IP_4_ as a substrate, but not 3,4,5,6-IP_4_; however a kinetic analysis of IPK1 with each IP_4_ was not performed (14). Our observations are consistent with the 1- and 3-phosphates stabilizing the bilobed structure of IPK1, thereby promoting its activation though recruitment of the N-lobe using a mechanism similar to that reported for protein kinases (12).

IPK1 was unable to use 1,3,4,5-IP_4_ as a substrate, which suggests that the 6-position may play a dual role in both IP binding and activation; abolishing both functions decreased its use as a substrate to levels below the detection limit of our assay. The *K_D_* values for 1,3,4,5-IP_4_ and 1,3,4,6-IP_4_ were similar; however, 1,3,4,6-IP_4_ could be used as a substrate, whereas 1,3,4,5-IP_4_ could not. This indicates that the decreased binding affinity of 1,3,4,5-IP_4_ for IPK1 was not by itself the underlying factor for its inability to be used as a substrate (Table 1). The 6-phosphate-binding site plays a key role in IPK1 activation by preventing clasp formation (a critical step in the IPK1 catalytic cycle) in the absence of IP substrate. Binding of the 6-phosphate to Lys-200 disrupts the interaction between Lys-200 and Glu-255, and this newly freed Glu-255 binds to Trp-129, thereby promoting clasp formation between the L3 loop and α6 helix (13). However, the K200A mutant, as well as other mutants that disrupted interaction with the 6-phosphate (K170A, N238A, and N239A), did not show any activity for 1,3,4,5-IP_4_, so intramolecular changes in the 6-phosphate-binding site are not essential for IPK1 activation (data not shown). It is possible that 1,3,4,5-IP_4_ adopts alternative binding orientations in which the 2′-hydroxyl is not accessible for phosphorylation (7, 15). Further experimentation will be required to ascertain why 1,3,4,5-IP_4_ displays no activity.

##### Critical Roles of Arg-130 and 1-Phosphate in IPK1 Activation

**FIGURE 3.**
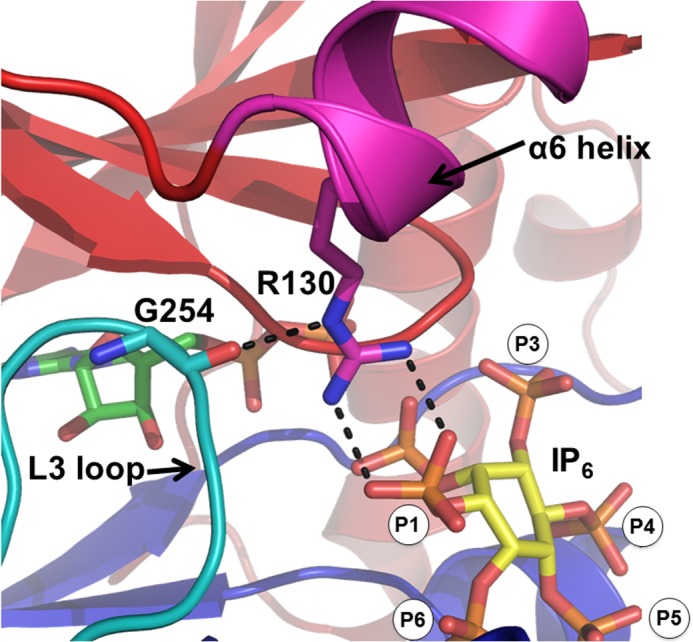
**Clasp formation between the α6 helix and L3 loop.** Interactions between Arg-130 (*magenta* stick form) and Gly-254 (*cyan* stick form) mediate clasp formation between the L3 loop (*cyan*) and α6 helix (*magenta*) of the C-lobe (*blue*) and of the N-lobe (*red*), respectively. Arg-130 interacts with the 1-phosphate of the IP, which is conducive to clasp formation. This image was created using PyMOL and a product-bound structure of IPK1 (Protein Data Bank code 3UDZ). ADP (*green*) and inositol 1,2,3,4,5,6-hexakisphosphate (*IP_6_*; *yellow*) are shown in stick form. *Dashed lines* indicate hydrogen bonds.

On the basis of previous crystal structures, we mutated each substrate-binding residue to alanine to determine the critical contacts between IPK1 and the IP. Each mutation abolished an interaction with one or two phosphates (Table 3), and we determined the *K_m_* and *k*_cat_ for each mutant in the presence of IP_5_. The impacts of the mutations and the activities of the IP_4_s were largely symmetric; abolishing single contacts with a phosphate group through mutation had a similar impact as removing the phosphate from the substrate. A notable difference between the IPK1 mutant and IP_4_ data was the asymmetric impact of abolishing the interaction with the 1-phosphate. Given that Arg-130, located on the N-lobe, is the only residue that interacts with the 1-phosphate, we expected that an IP lacking the 1-phosphate would correspond with the activity of this mutant. However, the R130A mutant displayed no detectable activity, whereas 3,4,5,6-IP_4_ could be used by the wild-type enzyme, albeit with low activity (Tables 2 and 3). Structures of IPK1 in complex with IP show that Arg-130 forms a bond with Gly-254, establishing an interaction between the L3 loop and α6 helix and promoting clasp formation (Fig. 3) (9). This interaction, and therefore clasp formation, cannot occur in the R130A mutant, rendering IPK1 inactive. Transient clasp formation can occur in wild-type IPK1, even in the absence of a 1-phosphate, which allows the use of 3,4,5,6-IP_4_ as a substrate with low activity. The 1-phosphate interaction with Arg-130 is conducive to clasp formation, thereby rendering IPK1 fully active (Fig. 3). Thus, the 1-phosphate stabilizes the active conformation of IPK1.

##### One Critical Residue at Each Phosphate-binding Site

There are 13 residues that interact with the IP in the IPK1 active site, either directly or indirectly through ordered water molecules, and each of these residues results in a bond to a phosphate group. For each phosphate of the IP, there is a contact residue that plays a critical role, and mutation of that residue abolishes activity. As discussed above, Arg-130 interacts with the 1-phosphate of the substrate and is a primary contact between the IP and the kinase N-lobe. Arg-130 is essential due to its structural role in stabilizing the active state and not exclusively for its role in binding the 1-phosphate, as 3,4,5,6-IP_4_ can bind and be recognized as a substrate. An IP lacking the 3-phosphate also displayed low activity; however, the R45A mutation, which disrupted the second contact between the IP and N-lobe, moderately impaired activity (Table 3). Thus, it appears that N-lobe interaction with IP is mediated primarily through Arg-130 and the 1-phosphate. Mutation of Lys-411, which interacts with both the 3- and 4-phosphates, was critical for activity, yet mutations of Arg-415 and Tyr-419, which mediate other interactions with the 4-phosphate, did not markedly affect IPK1 activity, nor did R45A, which is the second contact with the 3-phosphate, as described above (Table 3). Both the 5- and 6-phosphates bind to Lys-170, which is critical for activity; however, other 5-phosphate interactions with Arg-192 and His-196 and other 6-phosphate interactions with Lys-200, Asn-238, and Asn-239 are dispensable for activity. Wild-type IPK1 displayed activity with IPs lacking the 3- or 5-phosphate, yet mutations of Lys-170 and Lys-411 rendered IPK1 inactive, likely due to the fact that these residues bind more than one phosphate (Tables 2 and 3). Sequence alignments of IPK1 reveal that Arg-45, Arg-415, and Tyr-419 are the only inositide-binding residues that are not conserved among plant, human, rat, and yeast (8). Accordingly, R45A, R415A, and Y419A mutations retained at least 60% of wild-type activity. The conservation of Arg-130, but not Arg-45, suggests that N-lobe contact with the IP is mediated primarily through the 1-phosphate. In summary, our data indicate that each phosphate has a critical residue in the active site without which the enzyme cannot function, notably Arg-130, Lys-170, and Lys-411 (Table 3). Whether Lys-170 and Lys-411 impact binding affinity to the extent that substrate recognition cannot occur or if these residues play structural roles in shaping the binding site into an active conformation, as Arg-130 does, remains to be determined.

##### Model of IP-induced Stabilization as a Mechanism of Selectivity of IPs

Our previous crystal structures revealed that IPK1 possesses a stable C-lobe and a destabilized N-lobe in the absence of IP, such that the inositide-binding pocket remains partially formed (9). We proposed a model wherein IPK1 links its interactions with IP substrate phosphate groups to stabilization of the N- and C-lobes and kinase activation. The stability of the C-lobe in the absence of IPs suggested that the roles of 4-, 5-, and 6-phosphates were to mediate the initial contact of the IP with IPK1. Our present study reveals that the 5- and 6-phosphates impact binding affinity more than the 1- and 3-phosphates. Our model also proposed that the 1- and 3-phosphates were required for N-lobe stabilization, as they act to bridge the N-lobe with the C-lobe. In our present study, IPK1 displayed substantially lower *k*_cat_ values with 3,4,5,6-IP_4_ and 1,4,5,6-IP_4_ than with IP_5_ or an IP_4_ lacking the 5-phosphate. IP-free crystal structures of IPK1 reveal that the destabilization of the N-lobe is centered at Arg-130, which directly binds to the 1-phosphate of the IP substrate (9). The mutation of R130A impaired IPK1 activity, suggesting that the N-lobe is required to be stabilized for IPK1 activation, a key feature of kinase activation (12). In short, our study reveals specific roles for each of the IP phosphates, linking IPK1 substrate specificity to IPK1 stability.

##### Conclusions

In this work, we have demonstrated that the phosphate profile of IP_5_ is mechanistically linked to IPK1 activation. We have identified phosphates at the 1-, 3-, and 6-positions as the most important for activation of IPK1, whereas phosphates at the 5- and 6-positions are more important for binding than those at the 1- and 3-positions. Identification of the roles of the phosphates of the IP supports our proposed model of IPK1 substrate specificity and may provide a basis for selective targeting of IPK1, as similarity among IP substrates continues to hinder development of specific inhibitors for IPKs. Inhibition of IPK1 would prove valuable in the investigation of the roles of higher IPs whose production is dependent on inositol 1,2,3,4,5,6-hexakisphosphate, the product of IPK1, as well as functional roles of IPK1 in mammals.

## Footnotes

2 The abbreviations used are:

IP: inositol phosphate
IPK: IP kinase
IP_5_: inositol 1,3,4,5,6-pentakisphosphate
N-lobe: N-terminal lobe
C-lobe: C-terminal lobe
ITC: isothermal titration calorimetry
AMP-PNP: adenosine 5′-(β,γ-imido)triphosphate
IP_4_: inositol tetrakisphosphate.

## References

[1] Irvine R. F. Schell M. J. (2001) Back in the water: the return of the inositol phosphates. Nat. Rev. Mol. Cell Biol. 2, 327–338 11331907 10.1038/35073015

[2] Hanakahi L. A. Bartlet-Jones M. Chappell C. Pappin D. West S. C. (2000) Binding of inositol phosphate to DNA-PK and stimulation of double-strand break repair. Cell 102, 721–729 11030616 10.1016/s0092-8674(00)00061-1

[3] Hilton J. M. Plomann M. Ritter B. Modregger J. Freeman H. N. Falck J. R. Krishna U. M. Tobin A. B. (2001) Phosphorylation of a synaptic vesicle-associated protein by an inositol hexakisphosphate-regulated protein kinase. J. Biol. Chem. 276, 16341–16347 11278843 10.1074/jbc.M011122200

[4] Vajanaphanich M. Schultz C. Rudolf M. T. Wasserman M. Enyedi P. Craxton A. Shears S. B. Tsien R. Y. Barrett K. E. Traynor-Kaplan A. (1994) Long-term uncoupling of chloride secretion from intracellular calcium levels by Ins(3,4,5,6)P_4_. Nature 371, 711–714 7935818 10.1038/371711a0

[5] Shi Y. Azab A. N. Thompson M. N. Greenberg M. L. (2006) Inositol phosphates and phosphoinositides in health and disease. Subcell. Biochem. 39, 265–292 17121279 10.1007/0-387-27600-9_11

[6] González B. Schell M. J. Letcher A. J. Veprintsev D. B. Irvine R. F. Williams R. L. (2004) Structure of a human inositol 1,4,5-trisphosphate 3-kinase: substrate binding reveals why it is not a phosphoinositide 3-kinase. Mol. Cell 15, 689–701 15350214 10.1016/j.molcel.2004.08.004

[7] Miller G. J. Wilson M. P. Majerus P. W. Hurley J. H. (2005) Specificity determinants in inositol polyphosphate synthesis: crystal structure of inositol 1,3,4-trisphosphate 5/6-kinase. Mol. Cell 18, 201–212 15837423 10.1016/j.molcel.2005.03.016

[8] González B. Baños-Sanz J. I. Villate M. Brearley C. A. Sanz-Aparicio J. (2010) Inositol 1,3,4,5,6-pentakisphosphate 2-kinase is a distant IPK member with a singular inositide binding site for axial 2-OH recognition. Proc. Natl. Acad. Sci. U.S.A. 107, 9608–9613 20453199 10.1073/pnas.0912979107 PMC2906834

[9] Gosein V. Leung T. F. Krajden O. Miller G. J. (2012) Inositol phosphate-induced stabilization of inositol 1,3,4,5,6-pentakisphosphate 2-kinase and its role in substrate specificity. Protein Sci. 21, 737–742 22362712 10.1002/pro.2049 PMC3403471

[10] Knighton D. R. Zheng J. H. Ten Eyck L. F. Xuong N. H. Taylor S. S. Sowadski J. M. (1991) Structure of a peptide inhibitor bound to the catalytic subunit of cyclic adenosine monophosphate-dependent protein kinase. Science 253, 414–420 1862343 10.1126/science.1862343

[11] Ozkirimli E. Post C. B. (2006) Src kinase activation: a switched electrostatic network. Protein Sci. 15, 1051–1062 16597828 10.1110/ps.051999206 PMC2242506

[12] Sicheri F. Moarefi I. Kuriyan J. (1997) Crystal structure of the Src family tyrosine kinase Hck. Nature 385, 602–609 9024658 10.1038/385602a0

[13] Baños-Sanz J. I. Sanz-Aparicio J. Whitfield H. Hamilton C. Brearley C. A. González B. (2012) Conformational changes in inositol 1,3,4,5,6-pentakisphosphate 2-kinase upon substrate binding. Role of N-terminal lobe and enantiomeric substrate preference. J. Biol. Chem. 287, 29237–29249 22745128 10.1074/jbc.M112.363671 PMC3436203

[14] Sweetman D. Johnson S. Caddick S. E. Hanke D. E. Brearley C. A. (2006) Characterization of an *Arabidopsis* inositol 1,3,4,5,6-pentakisphosphate 2-kinase (AtIPK1). Biochem. J. 394, 95–103 16223361 10.1042/BJ20051331 PMC1386007

[15] Chamberlain P. P. Qian X. Stiles A. R. Cho J. Jones D. H. Lesley S. A. Grabau E. A. Shears S. B. Spraggon G. (2007) Integration of inositol phosphate signaling pathways via human ITPK1. J. Biol. Chem. 282, 28117–28125 17616525 10.1074/jbc.M703121200 PMC2244811

